# Impact of preoperative self‐expandable metal stent on benign hepaticojejunostomy anastomotic stricture after pancreaticoduodenectomy

**DOI:** 10.1002/deo2.307

**Published:** 2023-10-28

**Authors:** Takafumi Mie, Takashi Sasaki, Kosuke Kobayashi, Tsuyoshi Takeda, Takeshi Okamoto, Akiyoshi Kasuga, Yosuke Inoue, Yu Takahashi, Akio Saiura, Naoki Sasahira

**Affiliations:** ^1^ Department of Hepato‐Biliary‐Pancreatic Medicine Cancer Institute Hospital, Japanese Foundation for Cancer Research Tokyo Japan; ^2^ Division of Hepatobiliary and Pancreatic Surgery Cancer Institute Hospital, Japanese Foundation for Cancer Research Tokyo Japan; ^3^ Department of Hepatobiliary and Pancreatic Surgery Juntendo University School of Medicine Tokyo Japan

**Keywords:** pancreaticoduodenectomy, hepaticojejunostomy anastomotic stricture, balloon‐assisted enteroscopy, endoscopic retrograde cholangiopancreatography, self‐expandable metal stent

## Abstract

**Objectives:**

Hepaticojejunostomy anastomotic stricture (HJAS) is a serious adverse event of pancreaticoduodenectomy. Preoperative biliary drainage with a self‐expandable metal stent (SEMS) is often performed before pancreaticoduodenectomy. The purpose of this study is to evaluate the risk factors and impact of preoperative SEMS placement on developing benign HJAS after pancreaticoduodenectomy.

**Methods:**

We retrospectively analyzed consecutive patients who underwent pancreatoduodenectomy at our institution between July 2014 and June 2020. Risk factors for benign HJAS were identified using univariate and multivariate logistic regression analysis. We also compared outcomes of preoperative biliary drainage using SEMS and non‐SEMS.

**Results:**

Of the 626 included patients, benign HJAS occurred in 36 patients (5.8%). The median follow‐up time was 36.7 months (interquartile range, 25.4–57.4 months). Multivariate logistic regression analysis revealed that lack of preoperative biliary drainage, preoperative bile duct diameter <5 mm, and former or current smoking were independent predictors of benign HJAS. In the preoperative biliary drainage group, the rate of preoperative bile duct diameter <5 mm was significantly lower in the SEMS group than in the non‐SEMS group (2.0% vs. 12.8%, *p* = 0.04).

**Conclusions:**

Preoperative biliary drainage with SEMS may be useful to maintain bile duct diameter ≥5 mm and to reduce benign HJAS as a result.

## INTRODUCTION

Pancreaticoduodenectomy (PD) is a highly invasive operation for periampullary diseases. Advances in surgical techniques and perioperative management have reduced postoperative mortality.^1^, ^2^ Benign hepaticojejunostomy anastomotic stricture (HJAS) is one complication that can cause repeated cholangitis, obstructive jaundice, intrahepatic bile duct stones, and liver abscesses. Longer survival after PD has been reported to be associated with a higher incidence of benign HJAS.^3^

The usefulness of neoadjuvant therapy (NAT) for pancreatic cancer has been reported.^4^, ^5^ Periampullary diseases often present with obstructive jaundice or elevated liver enzymes and require biliary drainage before upfront surgery or NAT. Endoscopic biliary stenting is recommended to relieve distal bile duct obstruction.^6^ An ideal stent would not cause recurrent biliary obstruction during NAT and until surgery. Self‐expandable metal stents (SEMSs), recently becoming the standard for preoperative biliary drainage, have been reported to achieve longer time to recurrent biliary obstruction than plastic stents and can be deployed safely in patients undergoing NAT.^7^, ^8^

The incidence of benign HJAS is inversely correlated with preoperative bile duct diameter.^9^, ^10^, ^11^ Evidence on the relationship between preoperative biliary drainage and the incidence of benign HJAS is inconclusive, but no reports have examined preoperative SEMSs and the incidence of benign HJAS.^11^, ^12^, ^13^ The aim of this study was to evaluate the risk factors for developing benign HJAS and clarify the relationship between SEMSs in the preoperative setting and the incidence of benign HJAS after PD.

## MATERIALS AND METHODS

### Patients

We retrospectively analyzed consecutive patients who underwent PD at our institution between July 2014 and June 2020. We defined HJAS as a stricture of the hepaticojejunostomy anastomosis requiring intervention due to cholangitis, jaundice, intrahepatic bile duct stones, or elevated liver enzymes. We defined benign HJAS as HJAS without irregular mucosa on endoscopy, irregular stenosis of the bile duct on cholangiography, or findings suspicious of malignancy on computed tomography (CT). Patients with malignant HJAS, in‐hospital mortality after PD, and previous extrahepatic bile duct resection were excluded.^11^ In cases requiring preoperative biliary drainage, cases in which CT or magnetic resonance imaging (MRI) was not performed between stent deployment and surgery were excluded. Cases requiring preoperative biliary drainage were treated with SEMSs, plastic stents, endoscopic naso‐biliary drainage (ENBD), or percutaneous transhepatic biliary drainage (PTBD). SEMSs were used in cases expected to undergo NAT for pancreatic cancer, after early obstruction of plastic stents, and in unresectable periampullary lesions. The diameter of SEMSs used was generally 10 mm. Plastic stents with 7, 8.5, or 10 Fr, ENBD tubes with 5, 6, or 7 Fr, and PTBD tubes with 7, 8, or 10 Fr diameters were selected at the physician's discretion. In the preoperative setting, SEMSs were placed so that the proximal end was at least 2 cm distal to the hepatic hilum.

This study was conducted in accordance with the Declaration of Helsinki. The study was approved by the ethics committee of our institution (approval number: 2023‐GB‐036). Informed consent for this study was waived because of its retrospective nature. Consent was obtained for all procedures performed.

### Pancreaticoduodenectomy

PD was performed using standardized techniques at our institution. Dissection around the superior mesenteric artery is completed using a supracolic anterior artery–first approach with the dissection levels determined by tumor type. Our group previously described systematic dissection during PD using three different levels (levels 1, 2, and 3) around the artery.^14^, ^15^

After removal of the specimen, reconstruction is performed using a modified Child's method, and pancreatojejunostomy is performed in all cases. The hepaticojejunostomy anastomosis was performed using 5‐0 monofilament with interrupted suture, running suture, or combined interrupted suture for the posterior wall and running suture for the anterior wall, depending on the thickness and size of the common hepatic duct. For laparoscopic PD, all anastomoses including hepaticojejunostomy were performed through a small incision of approximately 6 cm, similar to open PD. In case of a small diameter of the common hepatic duct, interrupted suture for both posterior and anterior walls with 6‐0 monofilament was often performed. Lost stent or external stent for hepaticojejunostomy anastomosis were inserted at the surgeon's discretion.

### Follow‐up

Patients were followed up with laboratory studies every 1–3 months and contrast‐enhanced CT was performed every 3 months for 2 years and every 6 months thereafter for a total of 5 years after resection. In cases with suspected HJAS, endoscopic retrograde cholangiopancreatography (ERCP) with single‐balloon‐assisted enteroscopy (SBE) was performed. Two types of SBE were used during the study period (SIF‐Q260; working length, 2000 mm; channel diameter, 2.8 mm, and SIF‐H290S; working length, 1520 mm; channel diameter, 3.2 mm; Olympus Medical Systems). All procedures were performed by experts or by trainees under their direct guidance. Cases with benign HJAS were treated with balloon dilation with or without biliary stent placement. Follow‐up was conducted up to June 30, 2022.

### Evaluation

This study evaluated the rate of benign HJAS, time from surgery to benign HJAS and risk factors of benign HJAS. The final CT or MRI before surgery was used to determine preoperative bile duct diameter at the level of the hepaticojejunostomy anastomosis. In cases that required biliary drainage, pre‐drainage bile duct diameter was evaluated separately on CT or MRI before biliary drainage. Bile duct diameters were measured using axial images. Cases undergoing preoperative biliary drainage were evaluated further, dividing the patients into those treated with SEMSs versus other drainage methods (non‐SEMS). Stenoses were categorized into the following four groups: complete obstruction, severe stenosis, mild stenosis, and other (cases in which the HJAS could not be reached endoscopically or those with multiple bile duct orifices).^11^ Patency time was defined as the time from surgery to balloon dilation for benign HJAS or to the last follow‐up date. Patients were divided into the HJAS and non‐HJAS groups depending on the presence or absence of HJAS.

### Statistical analysis

Continuous variables are presented as medians (interquartile range) and were compared using the Mann‐Whitney *U* test. Categorical variables are described as absolute numbers (proportions) and were analyzed using the chi‐squared or Fisher's exact test. A *p‐*value <0.05 was considered statistically significant. Patency time was calculated with the Kaplan‐Meier method and compared using the log‐rank test. The cut‐off value of preoperative bile duct diameter for the occurrence of HJAS was calculated with the area under the receiver operating characteristic curves. Factors with *p‐*values <0.05 in univariate analysis were used in multivariate logistic regression analysis, and odds ratios (ORs) and 95% confidence intervals (CIs) were calculated. All statistical analyses were performed with EZR ver. 1.54.^16^

## RESULTS

### Patient characteristics

Six hundred ninety patients underwent PD. Thirty‐five cases with malignant HJAS, 23 cases with no CT or MRI after preoperative biliary drainage, five cases of in‐hospital mortality, and one case with a previous history of extrahepatic bile duct resection were excluded. Finally, 626 patients were included. The median follow‐up time was 36.7 months (interquartile range, 25.4–57.4 months). Benign HJAS was observed in 36 patients (5.8%). Baseline and perioperative characteristics are summarized in Table 1. The HJAS group had less non‐smokers, less preoperative biliary drainage, thinner preoperative bile ducts, and more hepaticojejunostomy anastomoses constructed with interrupted sutures than the non‐HJAS group. There were no differences in NAT rate, portal vein resection rate, rate of lost stents placement at the hepaticojejunostomy anastomosis, postoperative bile leakage and pancreatic fistula rate, or adjuvant chemotherapy rate between the two groups.

**TABLE 1:** Patient characteristics.

	HJAS *n* = 36	non‐HJAS *n* = 590	*p‐*value
Age, median (IQR), years	69 (61–72)	68 (60–74)	0.74
Sex, male, *n* (%)	26 (72.2%)	344 (58.3%)	0.12
Body mass index, median (IQR), kg/m^2^	22.4 (20.2–25.5)	21.9 (19.9–23.9)	0.16
Smoking, *n* (%)			0.04
Never	13 (36.1%)	320 (54.2%)	
Former or current	23 (63.9%)	270 (45.8%)	
Comorbidities, *n* (%)			
Diabetes mellitus	13 (36.1%)	159 (26.9%)	0.25
Cardiovascular disease (including hypertension)	11 (30.6%)	257 (43.6%)	0.17
History of abdominal surgery	4 (11.1%)	113 (19.2%)	0.28
Laboratory data before pancreaticoduodenectomy, median (IQR)			
Total bilirubin, mg/dL	0.6 (0.4–0.8)	0.6 (0.4–0.8)	0.48
Aspartate aminotransferase, IU/L	24 (21–29)	23 (19–31)	0.57
Alanine aminotransferase, IU/L	24 (18–37)	22 (16–34)	0.46
γ‐glutamyl transpeptidase, IU/L	32 (21–49)	41 (21–107)	0.09
Alkaline phosphatase, IU/L	216 (178–277)	245 (188–338)	0.05
Albumin, g/dL	3.9 (3.7–4.1)	3.9 (3.6–4.2)	0.58
C‐reactive protein, mg/dL	0.06 (0.04–0.24)	0.09 (0.04–0.25)	0.49
Preoperative biliary drainage, *n* (%)			<0.01
None	31 (86.1%)	318 (53.9%)	
PS/ENBD/PTBD	5 (13.9%)	223 (37.8%)	
SEMS	0 (0%)	49 (8.3%)	
Preoperative bile duct diameter, median (IQR), mm	4.0 (3.1–5.9)	6.3 (4.6–9.0)	<0.01
Preoperative chemotherapy, *n* (%)			
Neoadjuvant chemotherapy	4 (11.1%)	104 (17.6%)	0.49
Palliative chemotherapy (followed by conversion surgery)	1 (2.8%)	24 (4.1%)	>0.99
Operation time, median (IQR), min	475 (423–552)	487 (425–560)	0.82
Intraoperative blood loss, median (IQR), mL	485 (340–715)	500 (300–758)	0.88
Portal vein resection, *n* (%)	7 (19.4%)	203 (34.4%)	0.07
Type of surgery and digestive tract reconstruction, *n* (%)			0.79
Pancreaticoduodenectomy with Billroth‐II reconstruction	33 (91.7%)	522 (88.5%)	
Pancreaticoduodenectomy with Roux‐en‐Y reconstruction	3 (8.3%)	68 (11.5%)	
Approach method, *n* (%)			>0.99
Open approach	35 (97.2%)	572 (96.9%)	
Laparoscopy‐assisted approach	1 (2.8%)	18 (3.1%)	
Suture method, *n* (%)			0.06
Combination of running and interrupted suture	21 (58.3%)	446 (75.6%)	
Interrupted suture	15 (41.7%)	137 (23.2%)	
Running suture	0 (0%)	7 (1.2%)	
Lost or external stent for hepaticojejunostomy anastomosis, *n* (%)	34 (94.4%)	508 (86.1%)	0.25
Postoperative complications, *n* (%)			
Bile leakage	0 (0%)	9 (1.5%)	>0.99
Clinically relevant pancreatic fistula	10 (27.8%)	165 (28.0%)	>0.99
Delayed gastric emptying	3 (8.3%)	103 (17.5%)	0.25
Clavien‐Dindo classification ≥3	3 (8.3%)	78 (13.2%)	0.61
Postoperative diagnosis			0.36
Pancreatic cancer	16 (44.4%)	301 (51.0%)	
Bile duct cancer	1 (2.8%)	58 (9.8%)	
Ampullary cancer	2 (5.6%)	50 (8.5%)	
Duodenal cancer	2 (5.6%)	23 (3.9%)	
Intraductal papillary mucinous carcinoma	4 (11.1%)	31 (5.3%)	
Neuroendocrine tumor	1 (2.8%)	21 (3.6%)	
Other malignant disease	3 (8.3%)	30 (5.1%)	
Intraductal papillary mucinous adenoma	6 (16.7%)	59 (10.0%)	
Other benign disease	1 (2.8%)	17 (2.9%)	
Adjuvant therapy, *n* (%)	17 (47.2%)	283 (48.0%)	>0.99

Abbreviations: ENBD, endoscopic naso‐biliary drainage; HJAS, hepaticojejunostomy anastomosis stricture; IQR, interquartile range; PS, plastic stent; PTBD, percutaneous transhepatic biliary drainage; SEMS, self‐expandable metal stent; IU, international units.

### Risk factors for benign HJAS

**FIGURE 1:**
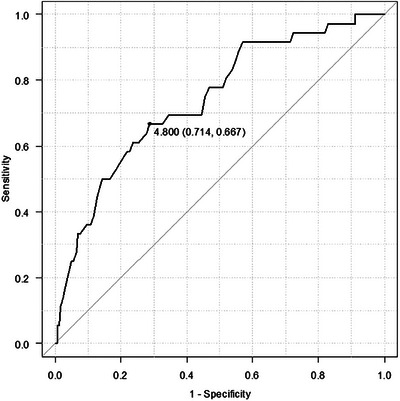
Receiver operating characteristic curve for hepaticojejunostomy anastomotic stricture based on preoperative bile duct diameter.

The median patency time among patients with HJAS was 17.6 months (95% CI, 13.8–25.1 months). The preoperative bile duct diameter was significantly shorter in the HJAS group than in the non‐HJAS group (*p* < 0.01). The cut‐off value of preoperative bile duct diameter was calculated to be 4.8 mm by receiver operating characteristic curve analysis, with an area under the curve of 0.741 (Figure 1). To be more clinically relevant, 5 mm was used as the cut‐off value. Table 2 shows univariate and multivariate analyses of factors associated with benign HJAS after PD. Cholangitis after preoperative stent or dysfunction of the preoperative stent was not associated with HJAS in univariate analysis. Lack of preoperative biliary drainage, preoperative bile duct diameter <5 mm, and interrupted sutures were predictors of benign HJAS. Interrupted suture was more common in cases with preoperative bile duct diameter <5 mm than with the other (37.8% vs. 17.9%, *p* < 0.01). Multivariate logistic regression analysis revealed that lack of preoperative biliary drainage (OR for HJAS, 2.96; 95% CI,1.04–8.40; *p* = 0.04), preoperative bile duct diameter <5 mm (OR, 2.93; 95% CI, 1.35–6.35; *p* < 0.01) and former or current smoking (OR, 2.10; 95% CI, 1.03–4.30; *p* = 0.04) were independent predictors for HJAS. In patients without preoperative biliary drainage, HJAS occurred in 9 of 178 cases with preoperative bile duct diameter ≥5 mm and in 22 of 171 cases with preoperative bile duct diameter < 5 mm (5.1% vs. 12.9%, *p* = 0.01).

**TABLE 2:** Univariate and multivariate analyses of risk factors for hepaticojejunostomy anastomosis stricture (HJAS).

		Univariate			Multivariate		
		**OR**	**95% CI**	***p‐*value**	**OR**	**95% CI**	***p‐*value**
Preoperative biliary drainage	not performed	5.30	2.03–13.80	<0.01	2.96	1.04–8.40	0.04
	performed	Ref					
Preoperative bile duct diameter	<5 mm	4.67	2.28–9.54	<0.01	2.93	1.35–6.35	<0.01
	≥5 mm	Ref					
Body mass index	≥22	1.45	0.73–2.86	0.29			
	<22	Ref					
Alkaline phosphatase	≥250 IU/L	0.54	0.26–1.09	0.09			
	<250 IU/L	Ref					
Cholangitis after preoperative drainage	Yes	0.83	0.09–7.56	0.87			
	No	Ref					
Dysfunction of preoperative drainage	Yes	1.82	0.30–11.10	0.87			
	No	Ref					
All interrupted suture	Yes	2.36	1.19–4.71	0.01	1.51	0.73–3.13	0.27
	No	Ref					
Operating time	≥480 min	0.90	0.46–1.76	0.75			
	<480 min	Ref					
Portal vein resection	Yes	0.46	0.20–1.07	0.07			
	No	Ref					
Malignant disease	Yes	0.61	0.26–1.45	0.26			
	No	Ref					
Postoperative pancreatic fistula	Yes	0.99	0.46–2.10	0.98			
	No	Ref					
Smoking	Former or current	2.10	1.04–4.22	0.04	2.10	1.03–4.30	0.04
	Never	Ref					

Abbreviations: CI, confidence interval; HJAS, hepaticojejunostomy anastomosis stricture; OR, odds ratio; Ref, reference.

### Subgroup analysis of the outcomes in preoperative biliary drainage

The incidence of benign HJAS in the SEMS group vs. the non‐SEMS group was examined in patients undergoing preoperative biliary drainage (Table 3). The SEMS group had lower hepatobiliary enzymes than the non‐SEMS group. There was no difference in preoperative bile duct diameter (8.9 mm vs. 8.2 mm, *p* = 0.19). There were no differences in adverse events after preoperative drainage and dysfunction of the preoperative stent. The SEMS group had a longer operating time, more intraoperative blood loss, a higher portal vein resection rate, and a higher rate of Billroth‐II reconstruction. There were no differences in suture methods or postoperative complications. Pancreatic cancer was more common and adjuvant therapy was conducted more frequently in the SEMS group.

**TABLE 3:** Characteristics of patients undergoing preoperative biliary drainage.

	SEMS *n* = 49	non‐SEMS *n* = 228	*p‐*value
Age, median (IQR), years	68 (60–73)	69 (62–75)	0.29
Sex, male, *n* (%)	27 (55.1%)	157 (61.4%)	0.43
BMI, median (IQR), kg/m^2^	21.5 (19.8–23.3)	21.7 (19.7–23.7)	0.97
Smoking, *n* (%)			0.88
Never	27 (55.1%)	123 (53.9%)	
Former or current	22 (44.9%)	105 (46.1%)	
Comorbidities, *n* (%)			
Diabetes mellitus	16 (32.7%)	64 (28.1%)	0.60
Cardiovascular disease (including hypertension)	22 (44.9%)	94 (41.2%)	0.64
History of abdominal surgery	7 (14.3%)	34 (14.9%)	>0.99
Laboratory data before pancreaticoduodenectomy, median (IQR)			
Total bilirubin, mg/dL	0.4 (0.3–0.6)	0.8 (0.5–1.3)	<0.01
Aspartate aminotransferase, IU/L	23 (19–29)	25 (21–40)	0.01
Alanine aminotransferase, IU/L	19 (15–28)	28 (20–60)	<0.01
γ‐glutamyl transpeptidase, IU/L	65 (30–155)	108 (57–237)	<0.01
Alkaline phosphatase, IU/L	269 (222–415)	326 (259–485)	0.03
Albumin, g/dL	3.7 (3.5–3.9)	3.7 (3.4–3.9)	0.63
C‐reactive protein, mg/dL	0.12 (0.08–0.24)	0.16 (0.06–0.55)	0.46
Preoperative biliary drainage method, *n*			
SEMS/Plastic stent/ENBD/PTBD	49/0/0/0	0/168/49/11	
Stent diameter, *n*	10mm/8mm/6mm: 46/1/ 2	PS: 7Fr/8.5Fr/10Fr: 86/74/8	
		ENBD: 5Fr/6Fr/7Fr: 5/3/41	
		PTBD: 7Fr/8Fr/10Fr: 2/5/4	
Stent diameter, median (range), French	30 (18–30)	7 (5–10)	<0.01
Pre‐drainage bile duct diameter, median (IQR), mm	13.0 (11.0–15.2)	13.6 (11.3–16.4)	0.17
Preoperative bile duct diameter, median (IQR), mm	8.9 (6.9–10.9)	8.2 (6.1–10.5)	0.15
Adverse events after preoperative drainage, *n* (%)			
Cholangitis	7 (14.3%)	57 (25.0%)	0.14
Pancreatitis	1 (2.0%)	11 (4.8%)	0.70
Cholecystitis	1 (2.0%)	2 (0.9%)	0.44
Asymptomatic elevated hepatobiliary enzymes	0 (0%)	10 (4.4%)	0.22
Dysfunction of the preoperative stent, *n* (%)	8 (16.3%)	67 (29.4%)	0.08
Preoperative therapy, *n* (%)			
Neo‐adjuvant	36 (73.5%)	31 (13.6%)	<0.01
Palliative chemotherapy (followed by conversion surgery)	7 (14.3%)	5 (2.2%)	<0.01
Operating time, median (IQR), min	546 (463–622)	490 (440–544)	<0.01
Intraoperative blood loss, median (IQR), mL	670 (500–990)	560 (370–835)	0.03
Portal vein resection, *n* (%)	38 (77.6%)	88 (38.6%)	<0.01
Type of surgery and digestive tract reconstruction, *n* (%)			0.03
Pancreaticoduodenectomy with Billroth‐II reconstruction	49 (100%)	208 (91.2%)	
Pancreaticoduodenectomy with Roux‐en‐Y reconstruction	0 (0%)	20 (8.8%)	
Approach method, *n* (%)			>0.99
Open approach	49 (100%)	228 (100%)	
Suture method, *n* (%)			0.23
Combination of running and interrupted suture	41 (83.7%)	200 (87.7%)	
Interrupted suture	8 (16.3%)	22 (9.6%)	
Running suture	0 (0%)	6 (2.6%)	
Lost or external stent for hepaticojejunostomy anastomosis, *n* (%)	34 (69.4%)	183 (80.2%)	0.13
Postoperative complications, *n* (%)			
Bile leakage	1 (2.0%)	2 (0.9%)	0.44
Clinically relevant pancreatic fistula	9 (18.4%)	71 (31.1%)	0.08
Delayed gastric emptying	4 (8.2%)	42 (18.4%)	0.09
Clavien‐Dindo classification ≥3	6 (12.2%)	31 (13.6%)	>0.99
Postoperative diagnosis			<0.01
Pancreatic cancer	44 (89.8%)	137 (60.1%)	
Bile duct cancer	4 (8.2%)	52 (23.1%)	
Ampullary cancer	1 (2.0%)	30 (13.2%)	
Duodenal cancer	0	0	
Intraductal papillary mucinous carcinoma	0	3 (1.3%)	
Neuroendocrine tumor	0	3 (1.3%)	
Other malignant disease	0	0	
Benign disease	0	3 (1.3%)	
Adjuvant therapy, *n* (%)	38 (77.6%)	134 (58.8%)	0.01

Abbreviations: ENBD, endoscopic naso‐biliary drainage; IQR, interquartile range; SEMS, self‐expandable metal stent; PTBD, percutaneous transhepatic biliary drainage; IU, international units.

**FIGURE 2:**
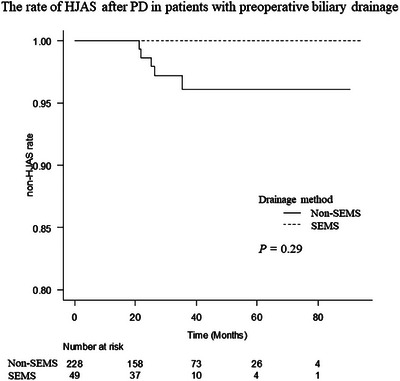
Rate of benign hepaticojejunostomy anastomotic stricture occurrence after pancreaticoduodenectomy in patients with preoperative biliary drainage. Benign hepaticojejunostomy anastomotic stricture occurred less often in the SEMS group than in the non‐SEMS group, but the difference was not significant. SEMS, self‐expandable metal stent

Median patency time was 28.0 months in the SEMS group and 29.3 months in the non‐SEMS group (*p* = 0.72). Benign HJAS occurred less often in the SEMS group than in the non‐SEMS group, but the difference was not significant (0% vs. 2.2%, *p* = 0.29; Figure 2). There were no differences in pre‐drainage and preoperative bile duct diameters between groups. There was also no difference in the rate of pre‐drainage bile duct diameter <5 mm between the two groups (0% in the SEMS group vs. 1.8% in the non‐SEMS group, *p* > 0.99). On the other hand, the rate of preoperative bile duct diameter <5 mm was significantly lower in the SEMS group (2.0% vs. 12.8%, *p* = 0.04; Table 4).

**TABLE 4:** Preoperative bile duct diameters

	SEMS *n* = 49	non‐SEMS *n* = 228	*p‐*value
Pre‐drainage bile duct <5 mm, *n* (%)	0 (0%)	4 (1.8%)	> 0.99
Preoperative bile duct <5 mm, *n* (%)	1 (2.0%)	29 (12.8%)	0.04

Abbreviation: SEMS, self‐expandable metal stent.

### Treatment for benign HJAS

The occurrence of HJAS was recognized as a result of cholangitis (72.2%), elevated hepatobiliary enzymes (22.2%), and intrahepatic stones (5.6%). SBE‐ERCP was attempted in all cases and was successful in 35 of 36 cases (97.2%). The one unsuccessful case underwent percutaneous balloon dilation for HJAS following PTBD because a guidewire could not be advanced through the hepaticojejunostomy anastomosis due to complete obstruction. Benign HJAS was treated with balloon dilation alone in most cases (97.2%), with both SEMS and plastic stent placement following balloon dilation in one case. Three of four patients with complete obstruction were successfully treated with SBE‐ERCP; one with SBE‐ERCP alone, one using the PTBD rendezvous technique and SBE‐ERCP, and one by puncturing the hepaticojejunostomy anastomosis with an injection needle followed by balloon dilation. No patients required surgical re‐anastomosis. (Table 5)

**TABLE 5:** Treatment for benign hepaticojejunostomy anastomotic stricture.

	*n* = 36
Reason for HJAS treatment, *n* (%)	
Cholangitis	26 (72.2%)
Elevated hepatobiliary enzymes	8 (22.2%)
Intrahepatic bile duct stone	2 (5.6%)
Approach, *n* (%)	
SBE‐ERCP	35 (97.2%)
PTBD	1 (2.8%)
Classification, *n* (%)	
Complete	4 (11.1%)
Severe	23 (63.9%)
Mild	9 (25.0%)
Treatment, *n* (%)	
Balloon dilation	35 (97.2%)
SEMS + plastic stent	1 (2.8%)
Recurrent HJAS, *n* (%)	14 (38.9%)

Abbreviations: ERCP, endoscopic retrograde cholangiopancreatography; HJAS, hepaticojejunostomy anastomotic stricture; SBE, single‐balloon‐assisted enteroscope; PTBD, percutaneous transhepatic biliary drainage; SEMS, self‐expandable metal stent.

## DISCUSSION

In this study, we retrospectively reviewed HJAS after PD. Benign HJAS was observed in 5.8% and the median patency time was 17.6 months. Lack of preoperative biliary drainage, preoperative bile duct diameter <5 mm, and former or current smoking were independent predictors of benign HJAS. The rate of preoperative bile duct diameter <5 mm was significantly lower in the SEMS group than in the non‐SEMS group (2.0% vs. 12.8%, *p* = 0.04).

The rate of benign HJAS and the time from PD to HJAS has been reported to be 2.6%–13.0% and 7.2–23.4 months, respectively, which are consistent with our results.^9^, ^10^, ^11^, ^12^, ^13^, ^17^ The rate of surgical re‐anastomosis was reported to be 4.8%–23.5%.^3^, ^9^, ^13^ However, with recent advances in balloon‐assisted enteroscopy, the surgical re‐anastomosis rate has decreased to 0%–7.5%, and none of the patients in this study required surgical re‐anastomoisis.^10^, ^11^, ^12^

Small preoperative bile duct diameter, high body mass index, preoperative biliary drainage, long operating time, postoperative pancreatic fistula, postoperative bile leak, benign lesions, and administration of adjuvant radiation therapy are reported risk factors for benign HJAS.^9^, ^10^, ^11^, ^12^, ^17^ In this study, lack of preoperative biliary drainage, preoperative bile duct diameter <5 mm, and former or current smoking were identified as independent risk factors for benign HJAS. The cut‐off value used for preoperative bile duct diameter varies from 4–8 mm in previous reports, but we used 5 mm based on our receiver operating characteristic curve.^9^, ^10^, ^11^ Large hepaticojejunostomy anastomoses are created more easily when the preoperative bile duct diameter is dilated, making benign HJAS less likely to occur. In the non‐preoperative biliary drainage group, the incidence of HJAS was significantly higher in patients with preoperative bile duct diameter <5 mm. A history of smoking is associated with severe postoperative complications.^18^ A history of smoking may make HJAS more likely due to microcirculatory disturbances. The use of running sutures for hepaticojejunostomy anastomosis with non‐dilated preoperative bile ducts is a reported risk factor for HJAS.^19^ The Suture method was not extracted as a risk factor in our study, as the hepaticojejunostomy anastomosis was formed by using an interrupted suture at the discretion of the surgeon when the preoperative bile duct was not dilated.

Pancreatic cancer is the most common reason for performing PD. The need for preoperative biliary drainage before PD remains controversial. However, cases requiring preoperative biliary drainage before PD have increased after NAT became the standard of care.^6^, ^20^ In a previous report, SEMSs had fewer biliary drainage‐related complications than plastic stents (24% vs. 46%, *p* = 0.011) in preoperative biliary drainage for pancreatic cancer.^21^ The stent dysfunction rate during NAT for pancreatic cancer has been reported to be significantly lower with SEMSs than with plastic stents (18.2% vs. 72.8%, *p* = 0.015).^7^ Perioperative complications have been reported to be similar between preoperative SEMS and plastic stent placement.^7^, ^21^, ^22^

Thus, the use of SEMSs in preoperative biliary drainage is increasing, but no reports have investigated the relationship between preoperative SEMSs and HJAS. In this study, none of the patients in the SEMS group developed benign HJAS. The bile duct diameter decreases after biliary drainage with plastic stents, but not with SEMSs, due to self‐expansion of the metal stent. Preoperative bile duct diameter <5 mm was significantly less common in the SEMS group (2.0% vs. 12.8%, *p* = 0.04). Preoperative SEMS placement may maintain bile duct diameter and facilitate the creation of hepaticojejunostomy anastomoses with large diameters.

The optimal diameter for the preoperative SEMSs remains controversial. The European Society of Gastrointestinal Endoscopy recommends using SEMSs with a 10 mm diameter for preoperative biliary drainage.^23^ However, pancreatitis and cholangitis are common adverse events that could delay surgery or NAT. In this study, there were no differences in pancreatitis, cholangitis, and stent dysfunction between the SEMS group and the non‐SEMS group. Recently, SEMSs with a 6 mm diameter have been reported to reduce pancreatitis and cholangitis while achieving patency time comparable to SEMSs with a 10 mm diameter.^24^ As residual pancreatic volume is associated with pancreatitis after SEMS deployment,^8^ 6 mm SEMSs may be preferable for preoperative biliary drainage in cases without pancreatic atrophy. Although 10 mm SEMSs were preferable to 6 mm SEMSs in maintaining the preoperative bile duct diameter ≥5 mm, there was a trade‐off between maintaining bile duct diameter and pancreatitis or cholangitis. The optimal preoperative SEMS diameter remains a topic for further research going forward.

In the past, benign HJAS was treated with surgical re‐anastomosis or PTBD.^3^, ^9^, ^13^ Recently, ERCP with balloon‐assisted enteroscope for HJAS achieved favorable results.^25^, ^26^, ^27^ In this study, initial treatment with SBE‐ERCP was successful in all but one case (97.2%). Balloon dilation with or without the deployment of plastic stents or SEMSs has been reported to be useful for benign HJAS.^28^, ^29^, ^30^ Despite the favorable outcomes with both plastic stents and SEMSs, one disadvantage is that additional procedures are required to remove stents. Balloon dilation without stent placement may be permissible in cases without a residual balloon notch, as recurrent HJAS is less common in this population.^30^

There are some limitations in this study. First, this is a retrospective study from a single institution. Despite a large sample size, there was a limited number of cases with benign HJAS. Second, the choice of surgical procedure and hepaticojejunostomy anastomosis suturing method were determined based on the discretion of the surgeon. Third, the impact of SEMS diameter on outcomes was not investigated.

In conclusion, we found that lack of preoperative biliary drainage, preoperative bile duct diameter <5 mm, and former or current smoking are independent risk factors for benign HJAS. Preoperative biliary drainage with SEMS may be useful to maintain bile duct diameter ≥5 mm and to reduce benign HJAS.

## CONFLICT OF INTEREST STATEMENT

None.
